# *β*-Glucan Subverts the Function of Myeloid Cells in Neonates

**DOI:** 10.1155/2024/2765001

**Published:** 2024-05-14

**Authors:** Yingying Chen, Hui Li, Lin Zhu, Quan Yang, Jie Zhou

**Affiliations:** ^1^Key Laboratory of Immunology, Sino-French Hoffmann Institute, School of Basic Medical Sciences, Guangdong Provincial Key Laboratory of Allergy and Clinical Immunology, The Second Affiliated Hospital, Guangzhou Medical University, Guangzhou 511436, China; ^2^Zhongshan School of Medicine, Sun Yat-sen University, Guangzhou 510000, China; ^3^Department of Clinical Laboratory, The Key Laboratory of Advanced Interdisciplinary Studies Center, The First Affiliated Hospital of Guangzhou Medical University, National Center for Respiratory Medicine, National Clinical Research Center for Respiratory Disease, Guangzhou, Guangdong 510120, China; ^4^Tianjin Institute of Immunology, Key Laboratory of Immune Microenvironment and Disease of the Ministry of Education, Department of Immunology, School of Basic Medical Sciences, Tianjin Medical University, Tianjin 300070, China

## Abstract

*β*-Glucan is the main component of the cell wall of pathogen-associated molecular patterns (PAMPs) including various yeast, fungi, or certain bacteria. Previous reports demonstrated that *β*-glucan was widely investigated as a potent immunomodulators to stimulate innate and adaptive immune responses, which indicated that it could be recommended as an effective adjuvant in immunotherapy. However, the detailed effects of *β*-glucan on neonatal immunity are still largely unknown. Here, we found that *β*-glucan did not affect the frequencies and numbers of myeloid cells in the spleen and bone marrow from neonates. Functional assay revealed that *β*-glucan from neonates compromised the immunosuppressive function of immature myeloid cells, which were myeloid-derived suppressor cells (MDSCs). Flow cytometry or gene expression analysis revealed that *β*-glucan-derived polymorphonuclear (PMN)-MDSCs produced lower level of reactive oxygen species (ROS) and arginase-1 (*Arg1*) in neonatal mice. Furthermore, *β*-glucan administration significantly decreased the frequency and ROS level of PMN-MDSCs in vitro. These observations suggest that *β*-glucan facilitates the maturation of myeloid cells in early life, which may contribute to its beneficial effects against immune disorders later in life.

## 1. Introduction

*β*-Glucan is a biologically active glucose polymer presenting in the cell wall of yeast, bacteria, fungi, edible mushrooms, cereal grains, oats, barley, wheat, and rye [1]. All *β*-glucans consist of glucose molecules linked together by a (1–3), (1, 4), or (1, 6) linear *β*-glycosidic chain; the types of glucans vary in length and branching structures occurring at different locations along the backbone of the polymer [2]. *β*-Glucan belongs to pathogen-associated molecular patterns (PAMPs) and possesses broad immunomodulatory function, including activation of innate immune functions and adaptive immunity [3–5]. *β*-Glucan activates innate immunity cells such as macrophages, dendritic cells, granulocytes, and natural killer (NK) cells, to enhance the production of chemokines and proinflammatory cytokines [6]. The ability of *β*-glucan to activate adaptive immunity cells, such as CD4^+^ or CD8^+^ T cells and B cells, is able to enhance the host's antitumor defense that is inhibiting the growth and metastasis of tumor [7, 8]. Despite the extensive studies on *β*-glucan-mediating immunomodulation, anti-inflammatory, and antitumor function, the detailed effect on neonatal immunity is not completely understood.

Myeloid-derived suppressor cells (MDSCs) are the accumulation of immature myeloid cells due to the perturbation of normal myeloid cells under certain pathological conditions such as tumor and infections [9]. MDSCs are mainly composed of two subsets: polymorphonuclear (PMN-MDSCs) and monocytic (M-MDSCs) [10]. PMN-MDSCs represent the dominant subset and preferentially utilize reactive oxygen species (ROS), peroxynitrite, and prostaglandin E2 (PGE2) to exert their immunosuppressive activity [10]. M-MDSCs exert immunosuppressive effects mainly through NO production and promoted higher expression of arginase-1 (ARG1) [10]. MDSCs have potent suppressive activity to inhibit T cell, B cell, or NK cell immune responses [11]. Recently, it is widely investigated that *β*-glucan exerts antitumor immune responses by regulating differentiation and immunosuppressive function of MDSCs [12–14]. The regulation of *β*-glucan on neonatal MDSCs and its responses in neonates remains poorly understood.

In this study, we found that *β*-glucan did not change the frequencies and numbers of myeloid cells in neonates but compromised the immunosuppressive function of MDSCs in neonates. Mechanistically, *β*-glucan-derived PMN-MDSCs produced lower levels of ROS and *Arg1* in neonatal mice. In vitro assay further demonstrated that *β*-glucan significantly decreased the frequency and ROS level of PMN-MDSCs. These observations suggest that *β*-glucan facilitates the maturation of myeloid cells in early life, which may contribute to its beneficial effects against immune disorders later in life.

## 2. Materials and Methods

### 2.1. Mice

The Laboratory Animal Center of Tianjin Medical University provided the C57BL/6 wild-type mice. All mice were housed and maintained in specific pathogen-free conditions, and all animal experiments were approved by the Institutional Animal Care and Use Committee at Tianjin Medical University.

### 2.2. *β*-Glucan Treatment

For in vivo studies, neonatal mice at postnatal days 3–4 were injected intraperitoneally (i.p.) with 0.5 mg of *β*-glucan (Sigma) in 50 *μ*l of PBS, and i.p. injection of PBS alone was performed as control according to a previous study [15]. For in vitro study, lin^−^ cells isolated from bone marrow cells of neonatal mice were cultured in RPMI 1640 medium (BI) supplemented with 10% fetal bovine serum, 20 ng/ml GM-CSF (Peprotech), and 50 *μ*M 2-mercaptoethanol, with or without *β*-glucan (5, 25, or 50 *μ*g/ml). Media were half changed on day 3, and cells were analyzed by flow cytometry on day 5.

### 2.3. Flow Cytometric Analysis and Sorting

Single-cell suspensions were stained with surface antibodies in cold FACS buffer (containing 1 × PBS supplemented with 1% FBS and 2 mM EDTA) for 30 min at 4°C, and red cells were removed using ACK lysing buffer. Flow cytometric analysis was performed by a CytoFLEX flow cytometer (Beckman Coulter). Flow cytometric sorting was performed by a MoFlo Astrios EQs flow cytometer cell sorter (Beckman Coulter). Data were analyzed by FlowJo V10 (TreeStar). Mouse MDSCs (CD11b^+^Gr1^+^), PMN-MDSCs (CD11b^+^Ly6G^+^Ly6C^lo^), and M-MDSCs (CD11b^+^Ly6G^−^Ly6C^high^) were analyzed by flow cytometer. After surface staining, LIVE/DEAD staining (Thermo Fisher) was used to exclude the dead cells. The following antimouse antibodies were purchased from eBioscience: biotin antimouse CD3 (clone 145-2C11), biotin antimouse CD4 (clone GK1.5), biotin antimouse CD8 (clone 53-6.7), biotin antimouse Gr1 (clone RB6-8C5), biotin antimouse CD11b (clone M1/70), biotin antimouse Ter119 (clone TER-119), biotin antimouse B220 (clone RA3-6B2), biotin antimouse NK1.1 (clone PK136), and APC streptavidin. The following antimouse antibodies were purchased from BioLegend: APC antimouse CD4 (clone GK1.5), APC/Cyanine7 antimouse CD8a (clone 53-6.7), FITC antimouse CD11b (clone M1/70), Brilliant Violet 421 antimouse CD11b (clone M1/70), PE antimouse Ly6G (clone 1A8), APC antimouse Ly6C (clone HK1.4), Percp-cy5.5 antimouse Ly6C (clone HK1.4), and PE antimouse Ly-6G/Ly-6C (Gr-1) (clone RB6-8C5).

Lin^−^ cells are remaining cells in the bone marrow (BM) after exclusion of CD3, CD4, CD8, Gr1, CD11b, Ter119, B220, and NK1.1 positive cells [16]. The flow cytometric analysis and sorting for lin^−^ cell in the BM from neonatal mice were performed: BM cells were stained with primary antibodies (biotin antimouse CD3, biotin antimouse CD4, biotin antimouse CD8, biotin antimouse Gr1, biotin antimouse CD11b, biotin antimouse Ter119, biotin antimouse B220, and biotin antimouse NK1.1) for 30 min at 4°C, and cells were then washed and restained with APC Streptavidin for 30 min at 4°C. Subsequently, cells were analyzed and sorted by flow cytometer.

### 2.4. RNA Sequencing (RNA-seq) Library Preparation and Data Analysis

Neonatal mice at postnatal days 3–4 were injected intraperitoneally (i.p.) with 0.5 mg of *β*-glucan. About 5 × 10^5^ PMN-MDSCs in the spleen were sorted from neonatal mice at day 3 after *β*-glucan administration. PMN-MDSCs were resuspended using TRIzol reagent (Thermo Fisher), and M-MuLV Reverse Transcriptase (RNase H) was used to perform the reverse transcription. According to the manufacturer's protocol, PCR was carried out with Phusion High-Fidelity DNA polymerase, universal PCR primers, and index primer. RNA-seq data were collected using Illumina NovaSeq with 2 × 150 bp paired-end run. DESeq2 was used to estimate the significance of differential expression genes (DEGs) between the two experimental groups. The resulting *P* values were adjusted using the Benjamini–Hochberg approach for controlling the false discovery rate. DEGs were defined with adjusted *P* values < 0.05 and an absolute value of log2 (foldchange) >1. The Kyoto Encyclopedia of Genes and Genomes (KEGG) and Gene Ontology (GO) analysis of DEGs was performed using clusterProfiler and with *P* value < 0.05 was regarded as significantly enriched by differentially expressed genes.

### 2.5. MDSC Function Assay

MDSC function assay was performed as previously described [17, 18]. Briefly, MDSCs in the spleen from neonatal mice were sorted. CD3^+^ T cells in the spleen from adult mice were labeled with 5- (and-6)-carboxyfluorescein diacetate and succinimidyl ester (CFSE, Thermo Fisher Scientific, 2 *μ*M), stimulated with anti-CD3-coated (5 *μ*g/ml, Invitrogen) plates and soluble anti-CD28 (1 *μ*g/ml, eBioscience) antibody, and cultured alone or with MDSCs at different ratios for 3 days. Cells were then stained with CD4 and CD8a antibodies, and T cell proliferation was analyzed by the intensity of CFSE fluorescence using a CytoFLEX flow cytometer.

### 2.6. Quantitative Real-Time PCR

Total RNA was extracted with TRIzol reagent (Thermo Fisher Scientific) according to the manufacturer's instructions. StarScript III cDNA synthesis kit (Genstar) was used to synthesize the cDNA. *β-Actin* was used as a reference gene for normalization. The primer sequences in the study were listed in the following page: qPCR assay of *β-actin* forward, 5′-GACGGCCAGGTCATCACTATTG-3′, qPCR assay of *β-actin* reverse, 5′-AGGAAGGCTGGAAAAGAGCC-3′; and qPCR assay of *Arg1* forward, 5′-CTCCAAGCCAAAGTCCTTAGAG-3′, qPCR assay of *Arg1* reverse, 5′-AGGAGCTGTCATTAGGGACATC-3′.

### 2.7. ROS Production

ROS production was analyzed with the oxidation-sensitive dye CM-H2DCFDA (Thermo Fisher Scientific). Cells were incubated in RPMI 1640 with 1 *μ*M CM-H2DCFDA at 37°C for 30 min and then stained with CD11b, Ly6G, and Ly6C antibodies at 4°C for 30 min. The MFI of ROS in the MDSCs from neonatal mice was analyzed by flow cytometry.

### 2.8. Statistics

Statistical analysis was analyzed with GraphPad Prism 8.0 software (GraphPad Software). For data with normal distributions, a two-tailed Student's *t* test was performed for comparing two groups, and one-way or two-way ANOVA followed by a Tukey–Kramer multiple comparisons test was performed for comparing three or more groups. For data not obeying normal distributions, a Mann–Whitney *U* test was performed. All data were displayed as mean ± SEM, and statistical significance was displayed as  ^*∗*^*P*  < 0.05,  ^*∗∗*^*P*  < 0.01, and  ^*∗∗∗*^*P*  < 0.001.

## 3. Results

### 3.1. *β*-Glucan Did Not Affect the Numbers of Newborn Myeloid Cells

**Figure 1:**
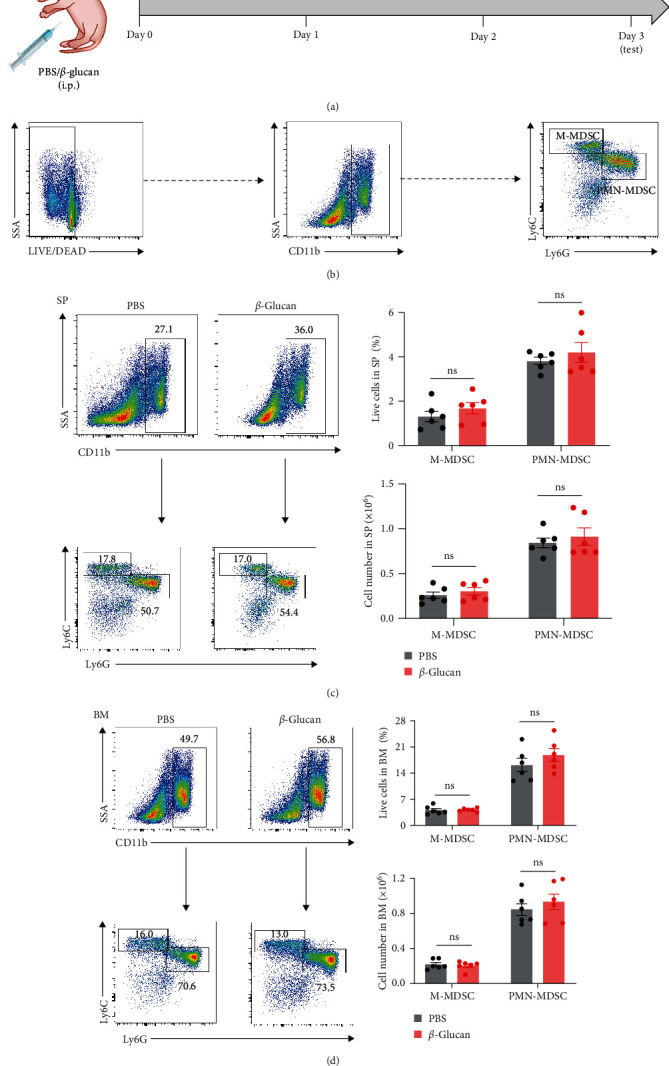
*β*-glucan did not affect the numbers of newborn myeloid cells. Neonatal mice at postnatal days 3–4 were injected intraperitoneally (i.p.) with 0.5 mg of *β*-glucan in 50 *μ*l of PBS, or with PBS as a control. Mice were sacrificed and cells were analyzed at day 3 after *β*-glucan treatment. (a) Experimental design. (b) The flow cytometric gating strategies for MDSCs were displayed. (c) Representative flow cytometric plots (left), and frequencies and numbers (right), of PMN-MDSCs and M-MDSCs in the spleen of neonatal mice after *β*-glucan treatment (*n* = 6). (d) Representative flow cytometric plots (left), and frequencies and numbers (right), of PMN-MDSCs and M-MDSCs in the bone marrow of neonatal mice after *β*-glucan treatment (*n* = 6). Data represented mean ± SEM from two independent experiments. ns, not significant.

To explore the effect of *β*-glucan on myeloid cells in neonatal mice, neonatal mice at postnatal days 3–4 were injected i.p. with 0.5 mg of *β*-glucan in 50 *μ*l of PBS, and i.p. injection of PBS alone was performed as control. After 3 days, the frequencies and numbers of myeloid cells from neonatal mice in the spleen and bone marrow were determined by flow cytometry (Figure 1(a)). The flow cytometric gating strategies for myeloid cells were showed in Figure1(b), including CD11b^+^Ly6G^−^Ly6C^high^ and CD11b^+^Ly6G^+^Ly6C^lo^ myeloid cells. It was showed that neonates with *β*-glucan administration displayed comparable levels of myeloid cells with controls in the spleen (Figure 1(c)). In addition, the frequencies and numbers of myeloid cells were comparable in the bone marrow between *β*-glucan challenged and control neonatal mice (Figure 1(d)). These observations suggested that *β*-glucan administration did not affect the frequencies and numbers of myeloid cells in neonates.

### 3.2. *β*-Glucan Compromised the Immunosuppressive Activity of MDSCs in Neonates

**Figure 2:**
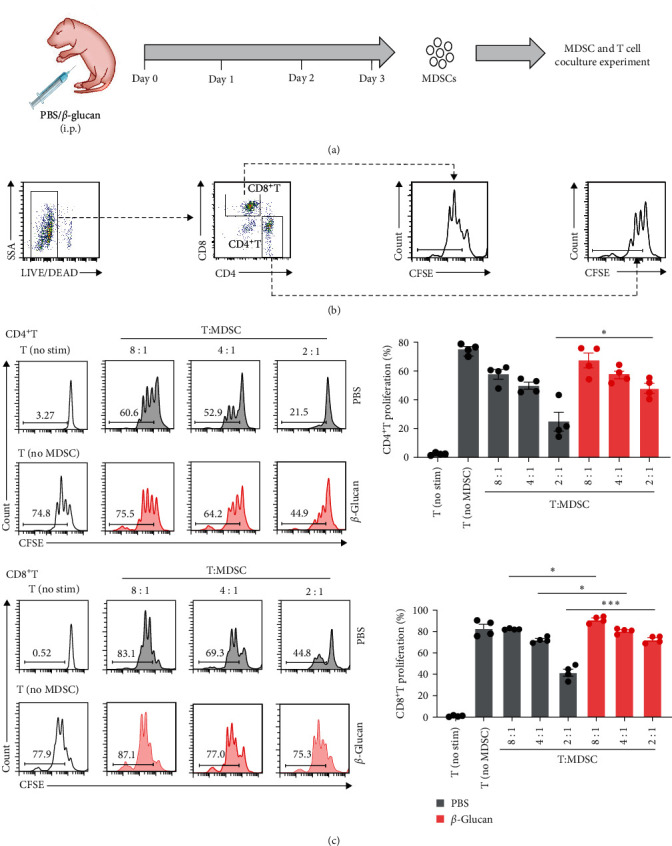
*β*-glucan compromised the immunosuppressive activity of MDSCs in neonates. (a) Experimental design of MDSC and T cell co-culture experiment. (b) The flow cytometric gating strategies for T cell proliferation. (c) CD3/CD28-inducible proliferation of CD4^+^ and CD8^+^ T cells in the presence of MDSCs. Non-stimulated T cells (no stim) and stimulated T cells without MDSCs were used as controls. T cell proliferation was evaluated using CFSE staining. Representative flow cytometry data (left) and statistical results (right) were shown (*n* = 4). Data represented mean ± SEM.  ^*∗*^*P*  < 0.05 and  ^*∗∗∗*^*P*  < 0.001.

Previous reports had investigated that splenic CD11b^+^Gr1^+^ myeloid cells from neonatal mice were immunosuppressive and were regarded as MDSCs [19, 20]. To further functionally determine the effect of *β*-glucan on myeloid cells in neonatal mice, CD11b^+^Gr1^+^ myeloid cells were isolated from the spleen in neonatal mice after *β*-glucan or PBS treatment, and a T cell coculture experiment was performed (Figure 2(a)). The flow cytometric gating strategies for T cell proliferation were showed in Figure 2(b). As expected, splenic CD11b^+^Gr1^+^ myeloid cells from PBS control displayed a dose-dependent immunosuppressive effect on T cells (Figure 2(c)), suggesting that they were MDSCs. However, the corresponding cells isolated from the spleen from *β*-glucan-treated neonatal mice apparently lowered the immunosuppressive activity (Figure 2(c)). These results indicated that *β*-glucan compromised the immunosuppressive function of MDSCs in neonates.

### 3.3. *β*-Glucan-Derived PMN-MDSCs Produced Lower Levels of ROS and Arg1

We next further investigated the functional mechanism of *β*-glucan-derived MDSCs.

**Figure 3:**
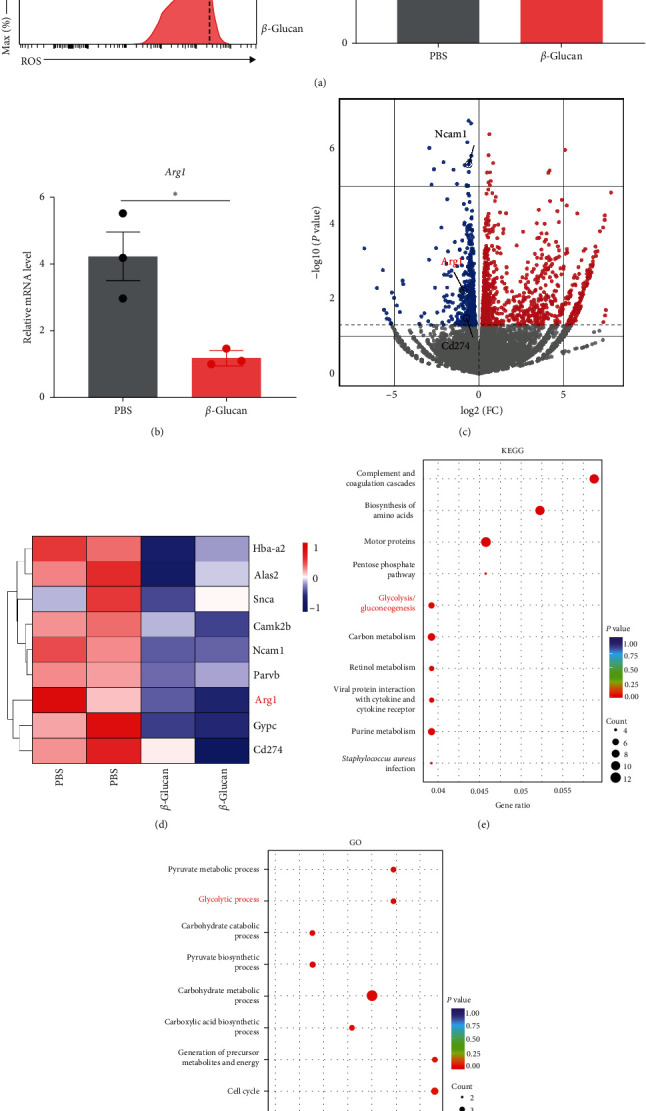
*β*-glucan-derived PMN-MDSCs produced lower levels of ROS and *Arg1*. Neonatal mice at postnatal days 3–4 were injected intraperitoneally with 0.5 mg of *β*-glucan in 50 *μ*l of PBS, or with PBS as a control. Mice were sacrificed and spleen cells were analyzed at day 3 after *β*-glucan treatment. (a) Representative flow cytometry histogram (left) and mean fluorescence intensity (MFI, right) of ROS expression in PMN-MDSCs at day 3 after *β*-glucan treatment (*n* = 6 from two independent experiments). (b) The expression of *Arg1* in PMN-MDSCs were measured by qRT-PCR (*n* = 3). (c) Volcano plot comparing differentially expressed genes from neonatal PMN-MDSCs, was analyzed using RNA-seq system. Red and blue dots represented up-regulated and down-regulated genes in *β*-glucan treatment PMN-MDSCs, and gray dots represented genes were not significant (*n* = 2). (d) Heatmap as determined by RNA-seq showing the selected gene expressions in the PMN-MDSCs from *β*-glucan treatment neonatal mice (*n* = 2). (e) The Kyoto Encyclopedia of genes and genomes (KEGG) plot showing the top enrichment pathways in PMN-MDSCs as determined by RNA-seq (*n* = 2). (f) gene ontology (GO) analysis of top expressed pathways in PMN-MDSCs as determined by RNA-seq (*n* = 2). Data represented mean ± SEM.  ^*∗*^*P*  < 0.05 and  ^*∗∗∗*^*P*  < 0.001.

Consistent with the observations of downregulated immunosuppressive function, *β*-glucan treatment caused the decrease of ROS (Figure 3(a)), an important mediator of immunosuppression in PMN-MDSCs under certain conditions [21]. In line with these results, a significant decrease of *Arg1* expression, a crucial regulator of immunosuppression in PMN-MDSCs [22], was observed in PMN-MDSCs after *β*-glucan treatment in neonatal mice (Figure 3(b)). To further investigate the mechanism underlying the regulatory effect of *β*-glucan on neonatal PMN-MDSCs, we performed RNA sequencing (RNA-seq) to evaluate the transcriptomes of splenic PMN-MDSCs from neonatal mice after PBS or *β*-glucan treatment. Among the total of 24,913 transcriptomes, 922 genes were significantly upregulated, and 522 genes were significantly downregulated in the *β*-glucan treatment PMN-MDSCs (Figures S1(a) and S1(b)). Similarly, volcano plot and heat plot analysis of RNA-seq displayed that mRNA transcripts of key immunosuppressive molecules, such as *Arg1*, *Cd274*, and *Ncam1* [22–24], were significantly downregulated in *β*-glucan-derived PMN-MDSCs (Figures 3(c) and 3(d)). However, RNA-seq revealed that mRNA transcripts of ROS production molecules, including *Ncf1*, *Ncf2*, *Ncf4*, *Cyba*, *Cybb*, and *Rac1*, were no changes between *β*-glucan- and PBS-derived PMN-MDSCs (Figure S1(c)), suggesting that the dysregulation of ROS in *β*-glucan-derived PMN-MDSCs occurred at post-transcriptional level. The Kyoto Encyclopedia of Genes and Genomes (KEGG) and Gene Ontology (GO) analysis of RNA-seq revealed that glycolytic process ranked as one of the most enriched pathways in PMN-MDSCs after *β*-glucan treatment (Figures 3(e) and 3(f)). Considering that the glycolysis pathway has been reported to regulate the immunosuppressive function of MDSCs [25, 26]. The precise signaling network that links the *β*-glucan, glycolysis, and immunosuppressive function of PMN-MDSCs in neonatal mice deserves further investigation.

### 3.4. *β*-Glucan Decreased the Percentage and ROS Level of Neonatal PMN-MDSCs In Vitro

**Figure 4:**
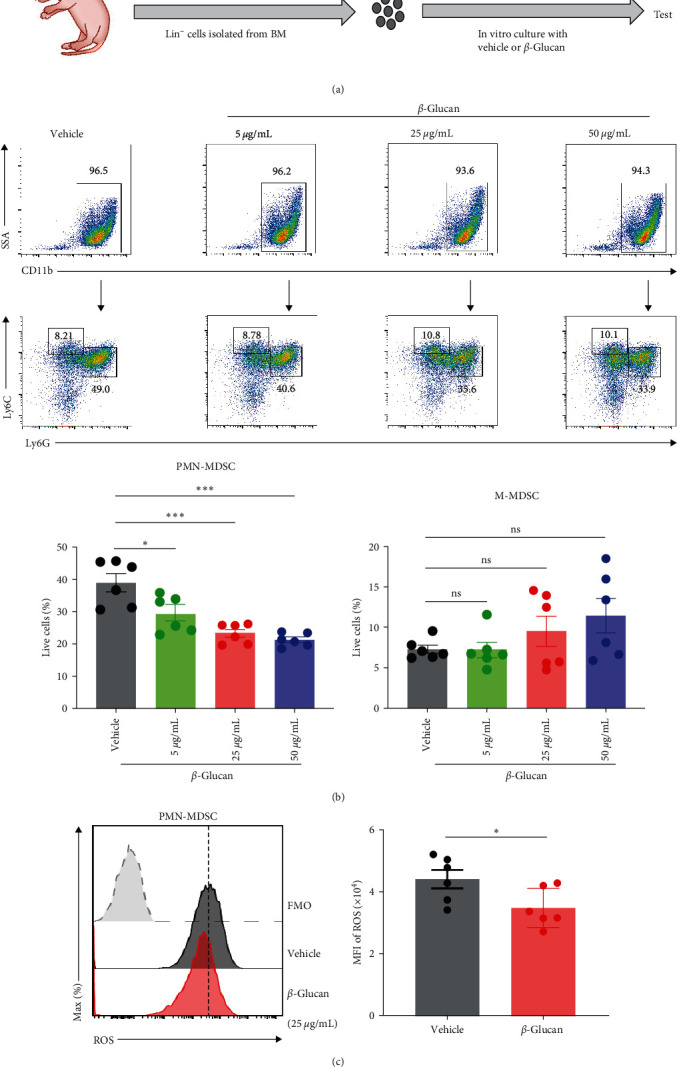
*β*-glucan decreased the percentage and ROS level of neonatal PMN-MDSCs *in vitro*. (a) Experimental design. (b) Representative flow cytometric plots (upper) and frequencies (lower) of PMN-MDSCs and M-MDSCs *in vitro* culture (*n* = 6). (c) Representative flow cytometry histogram (left) and MFI (right) of ROS expression in PMN-MDSCs *in vitro* culture (*n* = 6). Data represented mean ± SEM from two independent experiments.  ^*∗*^*P*  < 0.05,  ^*∗∗∗*^*P*  < 0.001 and ns, not significant.

To further investigate whether *β*-glucan influences MDSCs in vitro, we first cultured neonatal mice lin^−^ cells in the presence of GM-CSF with or without *β*-glucan. After 5 days, the frequencies and ROS level of MDSCs were evaluated by flow cytometry (Figure 4(a)). Results showed that *β*-glucan significantly decreased the frequency of PMN-MDSCs in a dose-dependent manner, whereas the frequency of M-MDSCs was not affected (Figure 4(b) and Figure S2(a)). We further analyzed the ROS level of PMN-MDSCs, and the data showed that a significantly reduction of ROS level was observed upon *β*-glucan administration in vitro (Figure 4(c)). Together, these results suggested that *β*-glucan could reduce the frequency and ROS level of PMN-MDSCs in vitro.

## 4. Discussion

Here, our study displayed that *β*-glucan administration significantly compromised the immunosuppressive function of MDSCs in neonates. The downregulation of ROS and *Arg1* in *β*-glucan-derived PMN-MDSCs might represent the functional mechanism. *β*-glucan administration also could reduce the frequency and ROS level of PMN-MDSCs in vitro culture. These observations indicated that *β*-glucan promoted the maturation of myeloid cells in early life, which might be helpful to resist the immune disorders later in life.

Our functional assay demonstrated that *β*-glucan administration compromised the immunosuppressive function of MDSCs in neonates. Previous studies had reported that the potential immunomodulation of MDSCs by *β*-glucan in the tumor microenvironment [12–14, 27]. Tian et al. [13] found that *β*-glucan treatment could promote the differentiation of M-MDSCs into a more mature CD11c^+^F4/80^+^Ly6C^low^ population via dectin-1 pathway in vitro and inhibit the suppressive function of M-MDSCs, leading to the delayed tumor progression. Albeituni et al. [14] demonstrated that yeast-derived particulate *β*-glucan (WGP) significantly decreased the accumulation and immunosuppressive function of PMN-MDSCs and reduced tumor weight and splenomegaly in tumor-bearing mice. Lo et al. [27] investigated that *β*-glucan could subvert the suppressive function of MDSC and augment antitumor immunity in oral squamous cell carcinoma (OSCC) patients. These findings on MDSCs' function were similar with our observations in *β*-glucan-challenged neonatal mice.

Our study found that *β*-glucan administration reduced ROS levels of PMN-MDSCs in vivo and in vitro culture. We also checked ROS levels of neonatal M-MDSCs in vivo and in vitro. Results showed that *β*-glucan treatment also decreased ROS levels of M-MDSCs in vivo and in vitro (Figure S3). ROS signaling is a central mediator of MDSC function and fate [21]. However, M-MDSCs exert immunosuppressive effects mainly through NO or ARG1 production and less through ROS pathway [28]. The mechanism of immunosuppressive function on M-MDSCs in neonatal mice after *β*-glucan treatment needs to be further explored.

*β*-glucan functioned as a PAMP, and several membrane-bound receptors including dectin-1, lactosylceramide, CR3, CD11b/CD18, and scavenger receptors involved in these immune responses following contact with *β*-glucan [29–33]. Dectin-1 is the most commonly studied *β*-glucan receptor and a type II transmembrane protein C-type lectin receptor, which results in the activation of the PI3K/Akt pathway and the phosphorylation of its intracellular immunoreceptor tyrosine-based activation motif [34, 35]. *β*-glucan binding to dectin-1 ultimately causes phagocytosis, microbial killing, and cytokine production [36, 37]. In addition, previous study and other findings highly suggested that *β*-glucan had great potential for activating dectin-1 on myeloid cells [13], which could mediate the immunosuppressive activity of MDSCs [38]. It is worthy of further investigation to explore the detailed mechanism underlying the effect of *β*-glucan on the immunosuppressive function of MDSCs in neonates.

## 5. Conclusion

In conclusion, our study showed that *β*-glucan apparently compromised the immunosuppressive function of MDSCs in neonates and promoted the maturation of myeloid cells in early life, which might be helpful to resist immune disorders later in life.

## Data Availability

The authors declare that all data supporting the findings of this study are available within the article or from the corresponding author on reasonable request.

## Supplementary Materials

Supplementary Materials Figure S1: mRNA transcripts of ROS production molecules on PMN-MDSCs. Figure S2: the flow cytometric gating for Lin-cells from neonatal mice. Figure S3: ROS levels of neonatal M-MDSCs in vivo and in vitro.
