# Supplementary material for: β-Glucan Subverts the Function of Myeloid Cells in Neonates
Source: J Immunol Res. 2024 May 14;2024:2765001. doi: 10.1155/2024/2765001 (PMC11108693; doi:10.1155/2024/2765001)
Supplement: Supplementary Materials — Figure S1: mRNA transcripts of ROS production molecules on PMN-MDSCs. Figure S2: the flow cytometric gating for Lin-cells from neonatal mice. Figure S3: ROS levels of neonatal M-MDSCs in vivo and in vitro. [file 2765001.f1.pdf]

Figure S1. mRNA transcripts of ROS production molecules on PMN-MDSCs

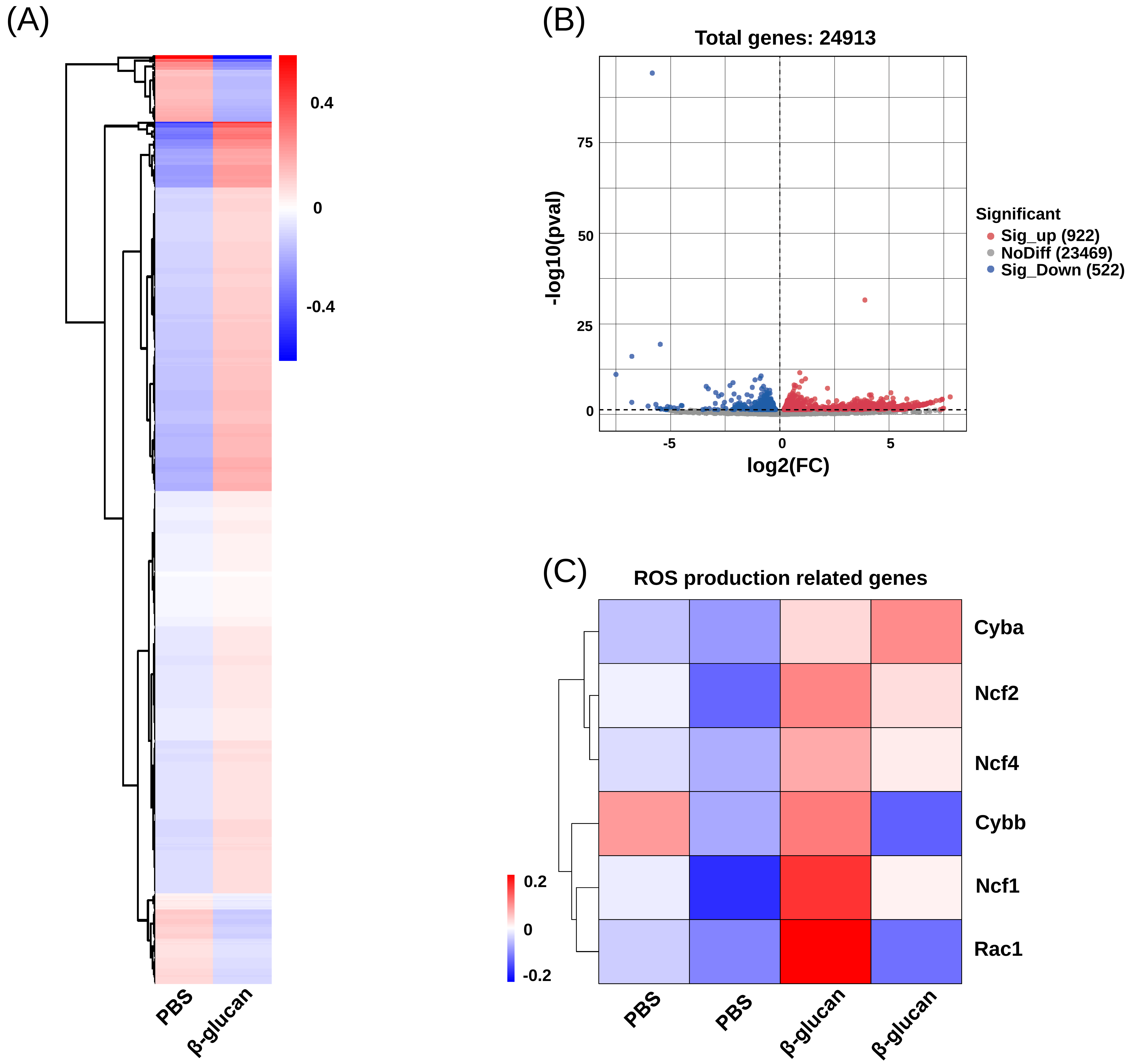

Fig. S1. mRNA transcripts of ROS production molecules on PMN-MDSCs. Neonatal mice at postnatal days 3-4 were injected intraperitoneally with 0.5 mg of β-glucan in 50ul of PBS, or with PBS as a control. Mice were sacrificed and splenic PMN-MDSCs were analyzed by RNA-seq at day 3 after β-glucan treatment (n = 2). (A) Heatmap showing the expression of genes in the PMN-MDSCs between PBS and β-glucan-treated groups. (B) Volcano plot comparing differentially expressed genes from neonatal PMN-MDSCs. Red and blue dots represented up-regulated and down-regulated genes in β-glucan-treated PMN-MDSCs, and gray dots represented genes were not significant. (C) Heatmap as determined by RNA-seq showing ROS gene expressions in the PMN-MDSCs from β-glucan-treated neonatal mice.

Figure S2. The flow cytometric gating for Lin^{-} cells from neonatal mice

(A)

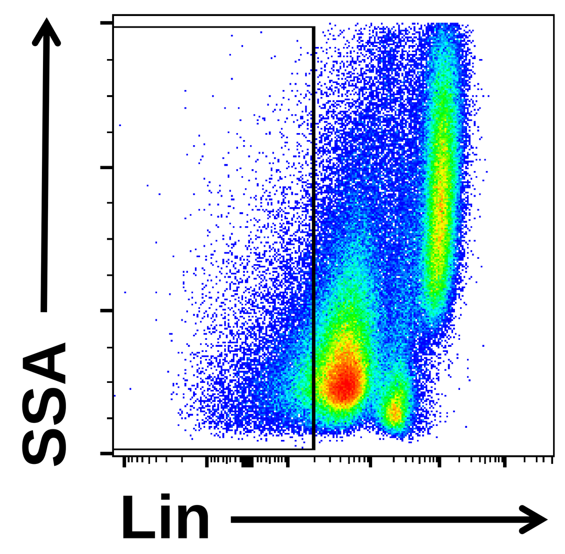

Fig. S2. The flow cytometric gating for Lin^{-} cells from neonatal mice. (A) The flow cytometric gating for Lin^{-} cells in the bone marrow from neonatal mice was displayed.

Figure S3. ROS levels of neonatal M-MDSCs *in vivo* and *in vitro*

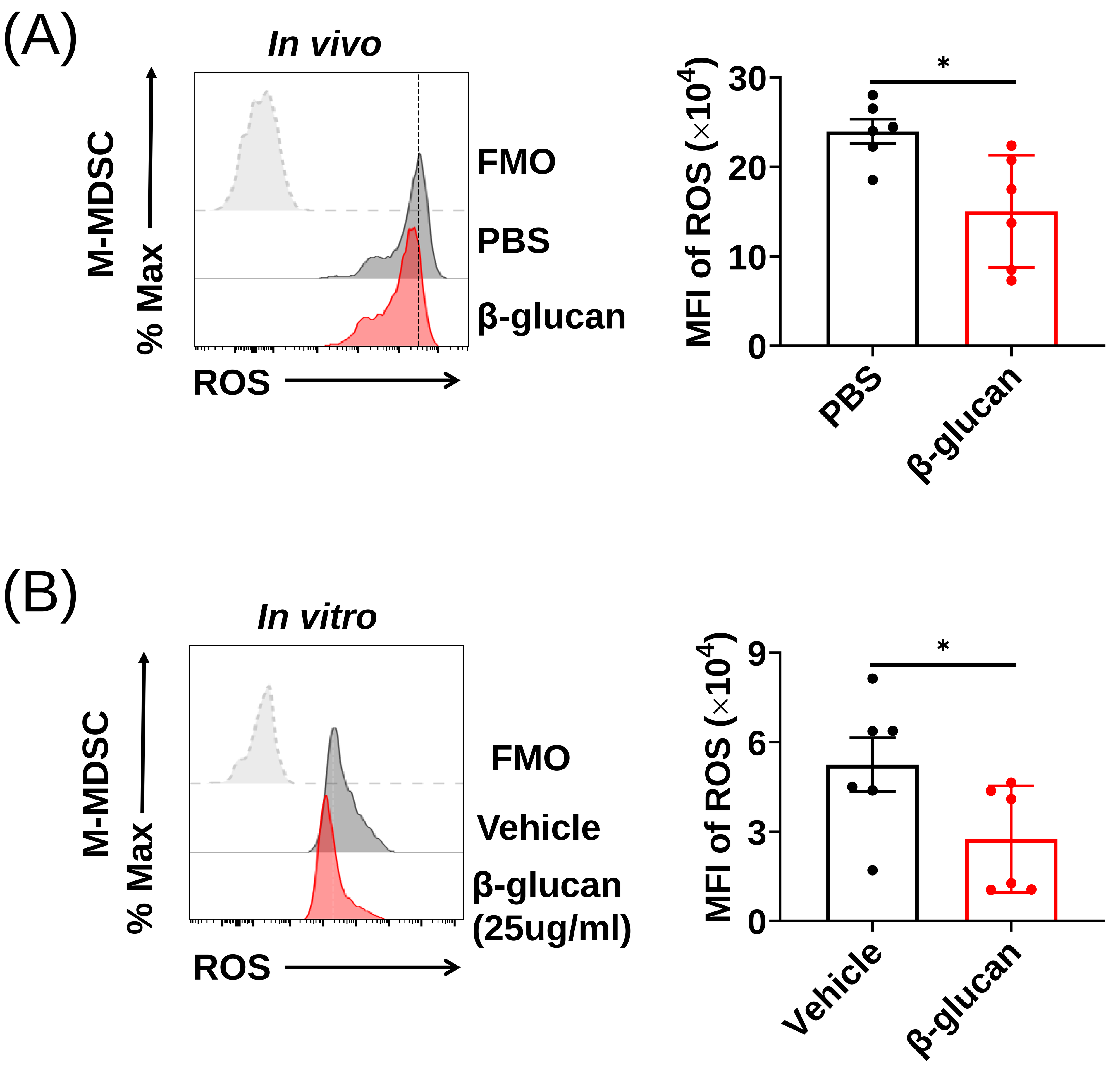

Fig. S3. ROS levels of neonatal M-MDSCs *in vivo* and *in vitro*. (A) Representative flow cytometry histogram (left) and mean fluorescence intensity (MFI, right) of ROS expression in M-MDSCs at day 3 after β-glucan treatment (n = 6). (B) Representative flow cytometry histogram (left) and MFI (right) of ROS expression in M-MDSCs *in vitro* culture (n = 6). Data represented mean ± SEM from two independent experiments. *P < 0.05.
